# Phenological Changes in the Fecal Microbiota of *Elaphurus davidianus* in Inner Mongolia Daqingshan National Nature Reserve

**DOI:** 10.3390/ani16111698

**Published:** 2026-06-01

**Authors:** Chunyan Liu, Jingjing Zhang, Yingshan Dong, Hua Ju, Taben Haoren, Lun He, Suritu, Haibo Ma, Jiawen Liu, Defu Hu, Dong Zhang, Liping Yan, Shumiao Zhang, Yunyun Gao

**Affiliations:** 1 School of Ecology and Nature Conservation, Beijing Forestry University, Beijing 100083, China; liuchunyan20001007@163.com (C.L.); jjzhang@bjfu.edu.cn (J.Z.); 18842558533@163.com (Y.D.); hudf@bjfu.edu.cn (D.H.); zhangdong_bjfu@bjfu.edu.cn (D.Z.); en130434@bjfu.edu.cn (Y.G.); 2 Inner Mongolia Daqingshan National Nature Reserve Administration, Hohhot 010010, China; 15034782871@163.com (H.J.); dqsxch@126.com (T.H.); 13804743238@163.com (S.); 13948191110@163.com (H.M.); 13604713365@163.com (J.L.); 3 Inner Mongolia Tongliao Forestry and Grassland Science Research Institute, Tongliao 028000, China; 4 China Wildlife Conservation Association, Beijing 100714, China; henry714@foxmail.com; 5 Beijing Milu Ecological Research Center, Beijing 100076, China

**Keywords:** 16s rDNA gene high-throughput sequencing technology, ex situ conservation, fecal microbiota, Père David’s deer, phenological changes, seasonal dynamics

## Abstract

Given the ongoing global decline in biodiversity, species reintroduction has become an important strategy for the conservation of endangered wildlife. Père David’s deer (*Elaphurus davidianus*), a historically extinct-in-the-wild species successfully reintroduced in China, represents a useful model for evaluating ecological responses following reintroduction into different habitats. Increasing evidence suggests that fecal microbiota can serve as a non-invasive indicator of host-associated microbial communities and their variation under different conditions. In this study, we investigated the fecal microbiota of a reintroduced population in the Daqingshan National Nature Reserve (Inner Mongolia). A total of 90 fresh fecal samples were collected between December 2021 and October 2023 across different seasonal periods. High-throughput 16S rRNA gene sequencing was used to characterize microbial community structure, and comparative analyses were conducted alongside published datasets from populations in Beijing and Shishou. The results revealed significant seasonal variation in microbial community composition and diversity, with distinct clustering of rainy, dry, and transitional (October) samples. Several taxa exhibited differential enrichment between seasons, suggesting temporal shifts in microbial community structure. A comparative analysis with other populations (Beijing and Shishou) revealed differences in microbial composition at broad taxonomic levels, suggesting potential variations related to regional environmental conditions, diet, and management practices. Overall, the observed patterns are consistent with seasonal variation in plant resource availability and highlight the potential influence of geographic context on microbial community structure. This study provides baseline data on the fecal microbiota of Père David’s deer in a semi-arid steppe environment and the potential indication of host suitability from the perspective of the microbiome.

## Simple Summary

Long-term monitoring of animal suitability is of great significance for ex situ conservation and species reintroduction. The fecal microbiota has become a core and reliable area for investigating animal ecology. We studied a group of Père David’s deer that were released into the Inner Mongolia Daqingshan National Nature Reserve. Over two years, we collected fresh fecal samples in both the rainy and dry seasons to analyze the bacteria in their feces. We found that the bacterial communities changed with the phenological period, helping the deer cope with different vegetation phenology throughout the year. We also observed that the fecal microbiota of this population differed from those reported in other regions, although the underlying causes of these differences cannot be determined based on the current data.

## 1. Introduction

Biodiversity is declining at an unprecedented rate [1], becoming one of the most significant global environmental crises [2]. Against this backdrop, species reintroduction has become a key conservation strategy for rescuing endangered wildlife and restoring their wild populations [3]. Although numerous reintroduction projects have been implemented worldwide, fewer than 50% have been successful [4,5,6,7]. The success of reintroduction depends not only on the recovery of the population but also on the ecological adaptability, reproduction, and health of individuals in new habitats [8]. Therefore, scientific assessment of the adaptation status of reintroduced individuals has become a core issue in optimizing conservation strategies and improving project effectiveness.

*Elaphurus davidianus* (Père David’s deer, also known as Milu), which belongs to the order Cetartiodactyla and family Cervidae, rated as extinct in the wild (EW) by the IUCN Red List in 2016, represents a typical example of successful global species reintroduction and population restoration [9]. Endemic to China, *E. davidianus* was historically widely distributed in the middle and lower reaches of the Yangtze River and the Yellow River in China [10]. This deer not only holds important cultural value but has also been considered an ecologically relevant species in wetland ecosystems. At the end of the 19th century, habitat destruction caused by human activities and overhunting led to the extinction of wild *E. davidianus* in China. Since 1985, when the Beijing Milu Ecological Research Center reintroduced 22 individuals from the United Kingdom [11], the population has gradually recovered, and multiple captive and wild populations have been established in Beijing, Hubei, and Jiangsu [12,13,14]. In 2021, 27 individuals were released into the Daqingshan National Nature Reserve (DNNR) in Inner Mongolia. The population is currently in good condition, comprising more than 60 individuals, and has begun natural reproduction [15], demonstrating suitability to the release environment. The expansion of the species from the original introduction sites to a northern semi-arid region with significant climatic and vegetation differences provides an opportunity to explore the suitability of different *E. davidianus* populations to heterogeneous habitats.

Due to its plasticity and close interaction with the host and environment, fecal microbiota has been widely used to characterize host-associated microbial communities and their variation in relation to diet and environmental factors [16,17]. The fecal microbiota serves as an important marker of host health status [18] and has been associated with host nutritional metabolism and immune function [19,20,21,22,23,24]. Chen et al. [25] and Liu et al. [26] observed seasonal fluctuations in microbial diversity and core genera in white-headed black langurs and rhesus macaques. Similarly, seasonal shifts in microbial community structure have been documented in other wild mammals, suggesting that microbiota may respond dynamically to environmental and dietary changes [27]. In addition, Bygrave et al. [28] observed lower lysozyme activity and higher bacterial species richness in urban finches, suggesting that environmental variation may be associated with shifts in host-associated microbiota.

With the continuous growth of reintroduced *E. davidianus* populations in China, increasing studies have focused on key issues in population recovery [29], such as housing management and pathogens, using fecal microbial communities as a tool [30,31]. Sun et al. [32] compared captive and wild populations and identified a shared core microbiome dominated by Firmicutes and Bacteroidetes, while revealing significant differences in community structure driven by diet, despite similar overall diversity. Zhang et al. [33] further demonstrated that habitat and dietary variation across ex situ populations significantly influence fecal microbial composition and diversity, highlighting the role of environmental factors. While these studies confirm the sensitivity of fecal microbiota to diet, environment, and health status, they largely rely on short-term or static observations. The long-term dynamics of the fecal microbiota during reintroduction, particularly under semi-arid steppe conditions characterized by greater climatic variability and limited supplementary feeding, remain insufficiently understood and warrant further investigation [34].

To fill this research gap, this study conducted a two-year continuous sampling of the *E. davidianus* population introduced into DNNR. Using 16s rDNA gene high-throughput sequencing technology, the fecal microbiota structure was characterized to explore its potential associations with seasonal environmental variation in the new habitat. We specifically tested the following hypotheses: (1) The composition and diversity of the fecal microbiota vary in response to seasonal changes in vegetation phenology. (2) Due to differences in climatic conditions and management regimes, the DNNR population exhibits distinct fecal microbial characteristics compared with other ex situ populations. This study provides baseline information on the fecal microbiota in a reintroduced population, which may be useful for future studies investigating host–microbiota relationships under different environmental conditions.

## 2. Materials and Methods

### 2.1. Research Area and Sample Collecting

The study was conducted in the Inner Mongolia DNNR, in a semi-arid typical steppe zone characterized by a continental semi-arid monsoon climate [35]. Based on climatic features and vegetation phenology, the annual cycle was divided into a rainy season (plant recovery and growth period, May–September) and a dry season (plant withering and dormancy period, October–April of the following year) [36,37,38]. The rainy season is marked by concentrated precipitation, warmer temperatures, and abundant high-quality plant resources, whereas the dry season features scarce precipitation, cold and arid conditions, and a decline in both the quantity and quality of available vegetation.

From December 2021 to October 2023, a total of 90 fresh fecal samples from *E. davidianus* were randomly collected across six sampling periods (Supplementary Table S1). Of these, 37 samples were assigned to the rainy-season group, whereas 53 samples were assigned to the dry-season group. As the plants are in the withering period from October to November, the October sample group is designated as a transitional group between the rainy and dry seasons. The imbalance in sample size among groups was primarily due to the inherent logistical constraints of field sampling, where collection schedules depended on site accessibility and the natural phenological cycles within the reserve. Nevertheless, the overall sample size in this study is relatively large compared with similar microbiome studies in wild animal populations [32,33]. Moreover, in subsequent analyses, robust statistical approaches (e.g., PERMANOVA) were employed to account for unequal sample sizes and to ensure the reliability of the results [39,40].

For each sampling event, the upper layer of fresh fecal piles, free from soil contamination, was collected into sterile centrifuge tubes, immediately labeled, and placed in a portable cooler with ice packs to maintain the samples at approximately 4 °C. Freshness was assessed based on the following criteria: a moist surface, intact pellet structure, absence of adhering soil or leaf litter, no visible insect colonization or fungal growth, and a strong odor characteristic of recent deposition [41]. Samples were transported to the laboratory within 4–6 h and subsequently stored at −20 °C until DNA extraction. To minimize the likelihood of collecting multiple samples from the same individual, sampling was restricted to freshly deposited feces within 3–4 h after defecation. Given the population size (>60 individuals) and the large spatial extent of the habitat, the probability of resampling the same individual within such a short time window is considered low. To reduce sampling bias, fecal samples were collected opportunistically from different individuals across the study area, with a spatial separation between samples to avoid repeated sampling of the same individual. Moreover, similar studies on wild ungulates using random fecal sampling have suggested that potential pseudoreplication does not substantially bias overall conclusions [42,43]. Nevertheless, as individual genetic identification was not performed, the possibility of pseudoreplication cannot be entirely excluded.

In parallel with the microbiome analysis, gastrointestinal parasites were also monitored in the same fecal samples using standard flotation and sedimentation techniques for fecal egg counts, as part of a complementary health assessment. The results indicated consistently low parasite burdens across seasons, with no clinical signs observed in the population. Detailed results are provided in Supplementary Table S2.

### 2.2. DNA Extraction and Polymerase Chain Reaction Amplification

DNA was extracted from fecal samples using the E.Z.N.A. Soil DNA Kit (Omega Bio-tek, Norcross, GA, USA) according to the manufacturer’s instructions. The V3–V4 variable region of 16s rDNA was amplified by polymerase chain reaction (PCR) with primer 338F-806R using the extracted DNA as a template [44]. Each PCR reaction contained 10 ng DNA, 4 µL 5× FastPfu Buffer, 2 µL 2.5 mM dNTPs, 0.8 µL each 5 µM forward and reverse primer, 0.4 µL FastPfu Polymerase, 0.2 µL bovine serum albumin, and ddH_2_O, for a final volume of 20 µL. The amplified products were visualized by 2% agarose gel electrophoresis, purified by PCR Cleanup Kit (Yuhua, Shanghai, China), and quantified by Qubit 4.0 (Thermo Fisher Scientific, Waltham, MA, USA).

### 2.3. Sequencing, Processing, and Statistical Analysis

For 16s rDNA amplicon sequencing, the purified amplicons underwent equimolar pooling and were paired-end sequenced on an Illumina Nextseq2000 platform (Illumina, San Diego, CA, USA), following the standard protocols of Majorbio Bio-Pharm Technology Co., Ltd. (Shanghai, China). The raw reads were subjected to quality filtering with Fastp (v0.19.6) to discard those with an average quality score below 20 or a length shorter than 50 bp. High-quality paired-end reads were subsequently assembled using FLASH (v1.2.7), requiring a minimum overlap of 10 bp. The merged sequences were then processed with Uparse (v7.1) for dereplication and clustering into operational taxonomic units (OTUs) at a 97% similarity threshold. In post-clustering curation, sequences originating from chloroplasts were removed from all samples. To ensure comparability in downstream diversity analyses, sequencing depth was normalized by rarefying the 16s rDNA gene sequences from each sample to 20,000 reads, which maintained an average Good’s coverage of 99.09%.

The taxonomy of each OTU representative sequence was analyzed by RDP Classifier version 2.2 against the 16s rDNA gene database (e.g., Silva v138) using a confidence threshold of 0.7. Within the Mothur platform (v1.30.1), rarefaction curves were generated, and a set of alpha diversity indices, encompassing observed OTUs, abundance-based coverage estimator (ACE), Chao1, Sobs, Shannon, Simpson, and Good’s coverage, was calculated. Differences in alpha diversity indices among groups were assessed using non-parametric tests (Wilcoxon rank-sum test for two-group comparisons or Kruskal–Wallis test for multiple groups). To evaluate beta diversity, principal coordinate analysis (PCoA) was performed based on Bray–Curtis dissimilarity matrices using the vegan package (v2.5–3) in R (version 4.4.0). In addition, permutational multivariate analysis of variance (PERMANOVA; Adonis function in vegan) was performed to test the statistical significance of differences in microbial community structure among groups. Differentially abundant bacterial taxa (from phylum to genus) across groups were identified via linear discriminant analysis effect size (LEfSe), applying significance thresholds of a linear discriminant analysis score > 2 and a *p* value < 0.05. Finally, the core microbiome across samples was characterized using the MicrobiomeAnalyst platform. Functional profiles of the microbial communities were predicted using PICRUSt2 based on 16S rRNA gene data. Predicted Kyoto Encyclopedia of Genes and Genomes (KEGG) pathways were summarized at Level 2, and their relative abundances across samples were visualized using heatmaps. Given the predictive nature of this approach, the functional results are presented in the Supplementary Materials.

Existing data on the fecal microbiota of *E. davidianus* populations from the Shishou Milu National Nature Reserve (Hubei Province, SS) and the Beijing Milu Park (Beijing, China) [33] were incorporated into this study. These datasets were obtained directly from the original authors. To ensure ecological comparability regarding seasonal timing, the fecal samples collected in March from the dry-season group were specifically selected for cross-regional comparison against the datasets from the Shishou Milu National Nature Reserve and the Beijing Milu Park. Based on the combined dataset, alpha diversity analysis, principal coordinate analysis (PCoA), Venn diagram analysis, taxonomic composition analysis, and linear discriminant analysis effect size (LEfSe) were performed to evaluate differences in microbial diversity, community structure, shared taxa, and differentially abundant features among populations.

## 3. Results

**Figure 1:**
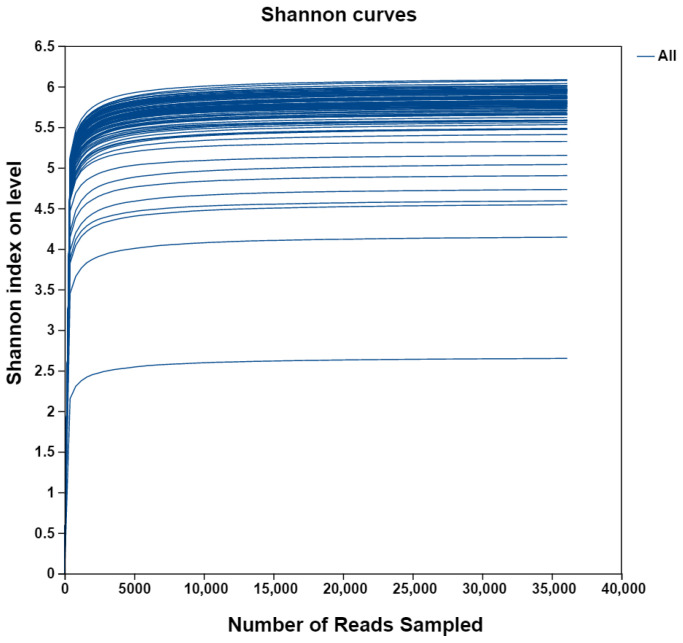
Rarefaction curve analysis of fecal samples from *Elaphurus davidianus* in the Daqingshan National Nature Reserve, Inner Mongolia.

All 90 fecal samples were subjected to high-throughput sequencing targeting the 16s rDNA gene. After quality filtering and assembly, a total of 8,370,604 high-quality sequences were obtained (Supplementary Table S3). The rarefaction curves approached a plateau, indicating that the sequencing depth was adequate (Figure 1). Clustering sequences at a 97% similarity threshold yielded 9381 OTUs, which were taxonomically assigned to 23 phyla, 52 classes, 128 orders, 239 families, and 514 genera.

**Figure 2:**
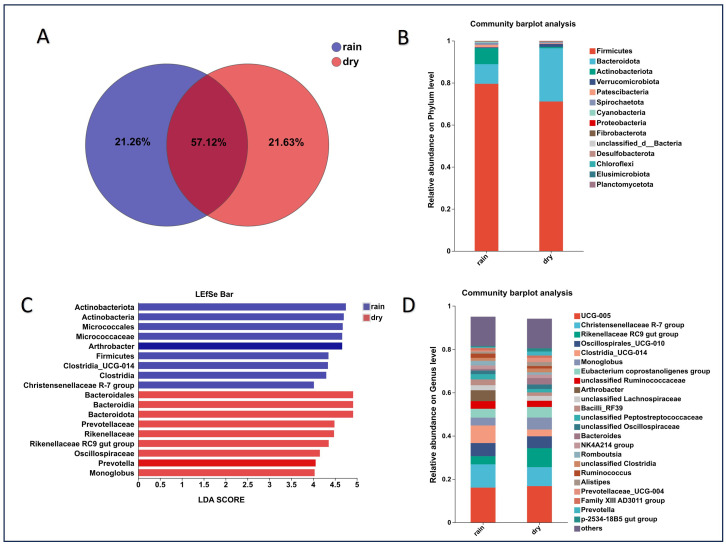
Composition of the fecal microbiota in *Elaphurus davidianus* during the rainy and dry seasons in the Daqingshan National Nature Reserve, Inner Mongolia: (**A**) Venn diagram showing shared and unique operational taxonomic units between seasons. (**B**) Relative abundance of bacterial communities at the phylum level. (**C**) Linear discriminant analysis effect size analysis identifying differentially abundant bacterial taxa from phylum to genus (linear discriminant analysis score > 2, *p* < 0.05). (**D**) Relative abundance of bacterial communities at the genus level.

In the rainy-season group, 19 phyla and 7387 OTUs were annotated. At the phylum level, Firmicutes was the most dominant phylum, accounting for 80% of the sequences, followed by Bacteroidetes (9%), Actinobacteria (8%), and Patescibacteria (1%). The dry-season group contained 23 annotated phyla and 7352 OTUs, with Firmicutes (71%) again being the dominant phylum, followed by Bacteroidetes (25%), Verrucomicrobiota (1%), and Actinobacteria (0.6%). A total of 5358 OTUs were shared between the two groups, representing 57% of all OTUs (Figure 2A). The dominance of Firmicutes in both groups is consistent with findings from other ruminant fecal microbiome studies [45] (Figure 2B). Compared with the dry-season group, the rainy-season group exhibited higher relative abundances of Firmicutes and Actinobacteria but lower abundances of Bacteroidetes and Verrucomicrobiota. Consequently, the Firmicutes/Bacteroidetes (F/B) ratio was higher in the rainy-season group.

At the genus level, 20 genera with a relative abundance greater than 1% were identified in the rainy-season group. The dominant genus was Oscillospiraceae_UCG-005 (16%), followed by the *Christensenellaceae R-7 group* (11%), Clostridia_UCG-014 (8%), Oscillospirales_UCG-010 (6%), *Arthrobacter* (5%), and *Eubacterium coprostanoligenes group* (4%). In the dry-season group, 24 genera exceeded the 1% abundance threshold. The dominant genus was Oscillospiraceae_UCG-005 (17%), followed by the *Christensenellaceae R-7 group* (9%), *Rikenellaceae RC9 gut group* (9%), *Monoglobus* (6%), Oscillospirales_UCG-010 (6%), and *Eubacterium coprostanoligenes group* (5%) (Figure 2D). Compared with the rainy-season group, the dry-season group harbored a greater number of genera above 1% abundance, with a notably higher abundance of the *Rikenellaceae RC9 gut group* and a lower abundance of *Arthrobacter*. LEfSe analysis identified the genus Clostridia_UCG-014 (Firmicutes) and *Arthrobacter* (Actinobacteria and Micrococcaceae) as signature taxa for the rainy-season group. In contrast, signature taxa for the dry-season group included the family Oscillospiraceae (Firmicutes), the genus *Prevotella* (Bacteroidetes), and the *Rikenellaceae RC9 gut group* (Bacteroidetes) (Figure 2C).

**Figure 3:**
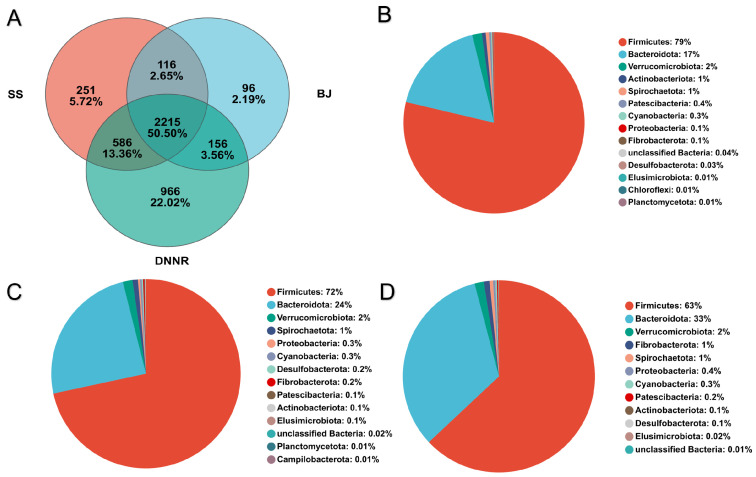
(**A**) Venn diagram showing shared and unique operational taxonomic units between regions. (**B**) Relative abundance of fecal microbiota at the phylum level of *Elaphurus davidianus* in Daqingshan National Nature Reserve, Inner Mongolia, in March. (**C**) Beijing Milu Park. (**D**) Shishou Milu National Nature Reserve, Hubei.

**Figure 4:**
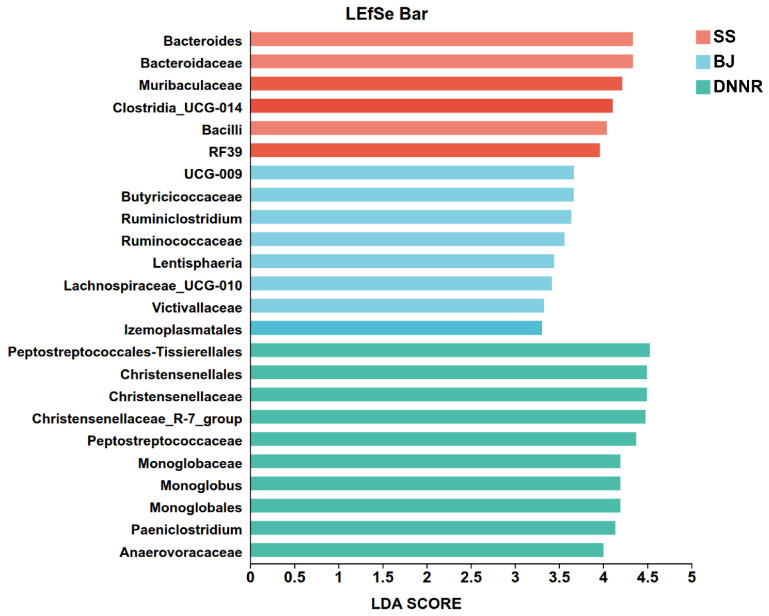
Linear discriminant analysis effect size (LEfSe) identifying differentially abundant bacterial taxa from phylum to genus among the Beijing, Shishou, and Inner Mongolia populations (linear discriminant analysis score > 2, *p* < 0.05).

Comparison of the intestinal microbiota of the DNNR population with those from the Shishou Milu National Nature Reserve and Beijing Milu Park revealed clear spatial variation in microbial community composition (Figure 3A). Both alpha diversity analyses and principal coordinate analysis (PCoA) indicated significant differences in microbial diversity and community structure among the three populations (Supplementary Figure S1; Supplementary Table S4). At the phylum level, all populations were dominated by Firmicutes and Bacteroidetes; however, their relative abundances differed among regions. The Beijing and DNNR populations were dominated by Firmicutes (72% and 79%, respectively), with lower proportions of Bacteroidota (24% and 17%, respectively) (Figure 3B,C). In contrast, the Shishou population exhibited a comparatively higher proportion of Bacteroidota (33%) and a lower proportion of Firmicutes (63%). Other phyla, including Verrucomicrobiota, Spirochaetota, and Actinobacteriota, were consistently present at low relative abundances across all populations, with only minor regional variation (Figure 3D). LEfSe analysis further identified differentially abundant taxa among the three populations (LDA > 2). LEfSe analysis identified the genus *Bacteroides* (Bacteroidaceae), as well as the family Muribaculaceae (Bacteroidota), as signature taxa for the SS group. In addition, the order Bacilli_RF39 (Bacilli) and the genus Clostridia_UCG-014 were also enriched in this group. In contrast, signature taxa for the BJ group included the genus *Ruminiclostridium* and the family Ruminococcaceae, together with the genus Butyricicoccaceae_UCG-009 (Butyricicoccaceae). Additional biomarkers were the genus Lachnospiraceae_UCG-010, the family Victivallaceae, the order Izemoplasmatales, and the class Lentisphaeria. For the DQS group, the dominant biomarkers comprised the genus Christensenellaceae_R-7_group (Christensenellaceae, Christensenellales) and *Monoglobus* (Monoglobaceae, Monoglobales). Similarly, the order Peptostreptococcales–Tissierellales and its corresponding family, Anaerovoracaceae, and the genus *Paeniclostridium* (Peptostreptococcaceae) (Figure 4).

**Figure 5:**
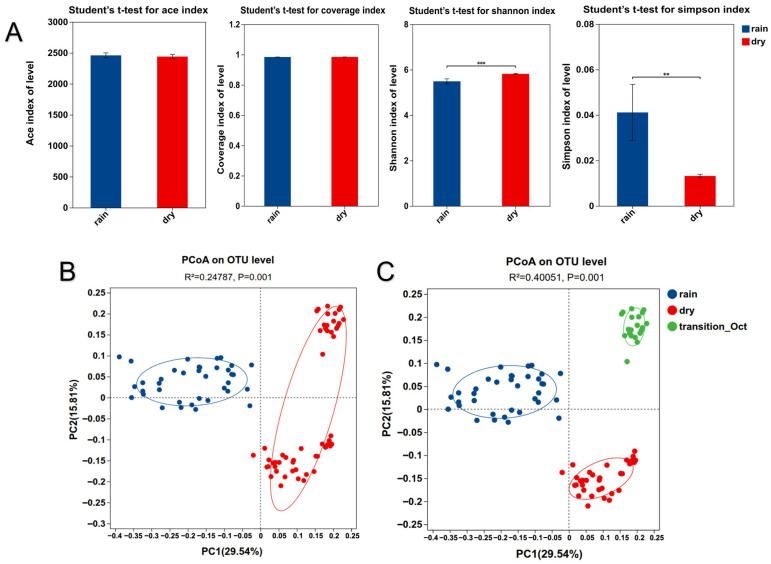
Diversity of the fecal microbiota in *Elaphurus davidianus* during the rainy and dry seasons in the Daqingshan National Nature Reserve, Inner Mongolia: (**A**) Box plots of alpha diversity indices (from left to right: abundance-based coverage estimator, Coverage, Shannon, and Simpson; **: *p* < 0.01; ***: *p* < 0.001) between the rainy- and dry-season groups. (**B**) Principal coordinates analysis (PCoA) plot based on Bray–Curtis distances for the rainy- and dry-season groups. (**C**) PCoA plot including the transitional (October) group alongside the rainy- and dry-season groups.

No significant differences were observed in species richness indices (e.g., ACE, Chao1, and Sobs) or in Good’s coverage index (Coverage) between the rainy- and dry-season groups (*p* > 0.05) (Figure 5A; Supplementary Table S5). However, significant differences were detected in diversity indices: The Simpson index was higher in the rainy-season group, while the Shannon index was higher in the dry-season group (Shannon: t = –3.452, *p* = 0.00086; Simpson: t = 2.717, *p* = 0.00794; see Supplementary Table S5 for full results) (Figure 5). These indices remained significant after false discovery rate (FDR) correction for multiple comparisons. Notably, the Shannon index is more sensitive to both species richness and evenness, placing greater weight on rare or low-abundance taxa, whereas the Simpson index is more influenced by dominant taxa and reflects the concentration of abundance within a few species [46,47,48]. Together, these results suggest that although overall microbial richness was comparable between seasons, community evenness and structure differed.

PCoA based on Bray–Curtis distances revealed a clear separation between the microbial community structures of the rainy- and dry-season groups, validating the seasonal grouping scheme (Figure 5B). This pattern was further confirmed by PERMANOVA (Adonis), which indicated that the differences between the rainy- and dry-season groups were statistically significant (R^2^ = 0.24787, *p* = 0.001). Notably, samples collected in October formed a distinct cluster separate from both the core rainy- and dry-season groups, supporting their designation as a transitional period (Figure 5C). Consistently, PERMANOVA analysis also demonstrated that the October group was significantly differentiated from the other groups (R^2^ = 0.40051, *p* = 0.001), further validating the effectiveness of the grouping strategy. Although the sampling spanned multiple years, visual inspection of the PCoA plots (Figure 5B,C) suggests that samples collected in the same season but from different years (e.g., March 2022 and March 2023) tended to cluster together. This pattern may indicate that seasonal variation plays an important role in shaping fecal microbial community structure during the study period, although potential interannual effects cannot be fully excluded.

## 4. Discussion

The responses of reintroduced endangered animal populations to novel environments can be reflected not only in population recovery but also in their persistence over time. As a well-documented example of species reintroduction, *E. davidianus* provides a useful system for examining ecological responses following translocation. The establishment of a wild population in the Daqingshan National Nature Reserve (DNNR) in recent years offers an opportunity to investigate population-level responses to ex situ conservation environments. In this study, high-throughput 16S rRNA gene sequencing was used to characterize the fecal microbial communities of *E. davidianus* during the initial years following reintroduction to DNNR. The aim was to describe patterns of variation in fecal microbiota structure and diversity in relation to seasonal phenological changes in a northern semi-arid grassland environment.

This study indicates that phenology is an important factor driving changes in the structure of animal fecal microbiota [27,49]. In fecal microbial diversity analysis, richness indices, including the Ace and Chao1 indices, showed no significant differences between the rainy- and dry-season groups. However, diversity indices, including the Shannon and Simpson indices, as well as the microbial community structure (Figure 5), changed significantly, indicating that the cyclical changes between rainy and dry seasons mainly reshaped community structure rather than altering the number of species. Notably, the F/B ratio in the rainy-season group was higher than that in the dry-season group (Figure 2B). Firmicutes, including *Clostridium* and *Lactobacillus*, dominate the decomposition of complex carbohydrates, producing short-chain fatty acids (SCFAs) and promoting energy absorption [50,51]; Bacteroidetes, which include *Bacteroides* and *Prevotella* [52], are adept at degrading plant polysaccharides and proteins [53,54]. The Firmicutes/Bacteroidetes ratio has been widely discussed in relation to energy metabolism in model organisms [55], with a higher F/B ratio generally indicating a higher weight gain rate in animals [56]. The higher F/B ratio in the rainy-season group suggests that energy metabolism during the rainy season is characterized by carbohydrate fermentation and energy storage. LEfSe multilevel species difference discrimination analysis (Figure 2C) revealed that *Arthrobacter* (Actinobacteria, Micrococcaceae) and Clostridia_UCG-014 (Firmicutes) were significantly enriched in the rainy-season group, whereas *Prevotella* (Bacteroidetes), *Rikenellaceae RC9 gut group*, and Oscillospiraceae (Firmicutes) were significantly enriched in the dry-season group. *Arthrobacter* has been reported to be involved in organic matter degradation in environmental systems [57], while Clostridia_UCG-014 participates in carbohydrate fermentation and is positively correlated with the production of beneficial SCFAs in the gut, such as butyrate, propionate, valerate, and caproate [58]. *Prevotella* and Oscillospiraceae promote the fermentation of dietary fiber and the production of SCFAs [59,60]. The *Rikenellaceae RC9 gut group* has been associated with lipid metabolism in some model systems and may have indirect effects on altering host backfat and muscle fatty acid composition [61].

During the vegetation recovery and growth season (May–September) in Inner Mongolia, which provides *E. davidianus* with carbohydrate-rich food, the microbial strategy favors efficient carbohydrate fermentation and energy storage. In contrast, during the dry season, cooling and frost cause plants to enter the withering and dormancy periods, leading the deer’s diet to shift to high-fiber dry grasses. To maximize energy acquisition from food, fiber-degrading bacteria are significantly enriched, and the microbial strategy shifts to plant fiber decomposition and lipid metabolism [62,63]. These strategies are consistent with previous observations of seasonal change in fecal microbiota in species such as Japanese macaques [64], indicating that phenological seasonal changes in fecal microbiota are also a mechanism for animals to cope with seasonal food resource pressures [65]. Importantly, the two diversity indices capture complementary aspects of these seasonal dynamics. The Simpson index reflects changes in dominant taxa and indicates greater dominance concentration in the rainy season, whereas the Shannon index is more sensitive to evenness and rare taxa, capturing increased community evenness in the dry season. Consistent with this pattern, the dry-season group was enriched in multiple fiber-degrading bacteria, such as *Prevotella* and the *Rikenellaceae RC9 gut group*, and exhibited higher Shannon diversity, indicating a more even distribution of taxa, including low-abundance species. In contrast, the rainy-season group was dominated by a few highly abundant taxa, such as *Arthrobacter* and *Clostridium*, and showed higher Simpson diversity, reflecting stronger dominance by a limited number of taxa.

The findings of this study suggest that geographic differences [66] and management practices [67] may contribute to variation in fecal microbiota among wildlife populations. LEfSe analysis identified distinct sets of differentially abundant taxa among the three populations. In the Shishou (SS) population, taxa such as *Bacteroides* and members of Muribaculaceae were significantly enriched. *Bacteroides* are closely associated with the degradation of starch-derived polysaccharides [68], whereas Muribaculaceae are specialized in fermenting complex polysaccharides with the capacity to produce propionate [69]. In addition, the enrichment of Clostridia_UCG-014 and members of Bacilli_RF39 further supports the potential importance of glycan utilization and diverse fermentation pathways in this population. Bacilli_RF39 has been reported as a saccharolytic, acetate-producing group [70], while Clostridia_UCG-014 has been associated with the degradation of complex dietary glycans and fermentation processes in intestinal microbiota studies [71]. Together, these taxa may reflect adaptation to relatively abundant and diverse plant-derived food resources under subtropical environmental conditions. In contrast, the Beijing (BJ) population was characterized by the enrichment of several taxa associated with fiber degradation and SCFA production, including *Ruminiclostridium*, Ruminococcaceae, Victivallaceae, and Lachnospiraceae_UCG-010 [68,72,73,74]. These groups are known to encode a wide range of carbohydrate-active enzymes involved in the breakdown of cellulose, hemicellulose, and other plant structural components. Other taxa, such as Butyricicoccaceae_UCG-009, Lentisphaeria, and Izemoplasmatales, have been associated with high-protein diets and may contribute to promoting host growth performance [75,76,77]. The presence of these taxa may be related to the combined effects of natural forage and supplementary feeding in managed environments, although direct causal relationships cannot be established in this study. For the Daqingshan (DNNR) population, characteristic taxa included Christensenellaceae_R-7_group, Monoglobus, and members of Peptostreptococcales–Tissierellales, including Anaerovoracaceae and Paeniclostridium. Monoglobus is known as a specialized pectin-degrading bacterium, suggesting an ability to utilize specific plant cell wall components [78,79], while Anaerovoracaceae has been associated with the utilization of diverse organic substrates and may be linked to improved feed efficiency in herbivores [80,81]. The enrichment of these taxa may reflect microbial adaptation to a relatively resource-limited, high-fiber diet under semi-arid conditions. *Paeniclostridium*, particularly *P. sordellii*, is generally regarded as an opportunistic pathogen [82,83]. Previous studies have shown that severe infections are typically associated with toxin-producing strains and occur under specific conditions [84,85], including tissue injury [86], compromised host immunity [87], polymicrobial infections, and microbiota dysbiosis [88]. These factors may further facilitate its proliferation and enhance its pathogenic potential.

Previous studies have shown that the fecal microbiota of reintroduced *E. davidianus* varies across geographic regions, with differences in habitat characteristics and management practices playing a role. These studies suggest that the compositional structure of fecal microbiota in different regions may reflect ecological niche replacement of dominant taxa, driven by variations in habitat and diet [89,90]. In the case of the populations from the DNNR and Shishou Milu National Nature Reserve, geographic variation may contribute to the observed differences. The Shishou Milu National Nature Reserve, located in a subtropical monsoon climate, offers more abundant food resources for *E. davidianus* during March compared to the phenological conditions in Inner Mongolia [91]. In contrast, environmental conditions in Daqingshan are characterized by a semi-arid climate, which may constrain both the availability and diversity of forage. Dietary differences may contribute to the observed regional variation in fecal microbiota. In the Daqingshan population, a detailed dietary survey [15] was conducted using the cage method and recorded 24 plant species from 19 genera and 10 families, with Poaceae and Asteraceae as the primary food sources. Although detailed seasonal dietary data are not available, these findings provide a general context for interpreting microbiota variation among populations. However, the lack of concurrent dietary data in this study limits our ability to directly link microbial patterns to specific dietary factors. In Shishou, stable isotope analysis has shown that *E. davidianus* primarily consumes Poaceae and Cyperaceae, with clear seasonal variation in response to plant availability [92]. In the Beijing Milu Park, *E. davidianus* are maintained under intensive management conditions, including captivity and supplementary feeding, whereas the DNNR population is free-ranging and relies primarily on natural forage. Although Beijing and Daqingshan (Inner Mongolia) share broadly similar vegetation types, as reflected in diets including alfalfa (*Medicago sativa*), green foxtail (*Setaria viridis*), and other herbaceous plants [93], differences in management practices may contribute to the observed variation in fecal microbiota. In particular, individuals in Beijing are typically maintained under more intensive management conditions, including captivity and supplementary feeding, whereas populations in Daqingshan experience more natural foraging conditions with limited human intervention. In this study, we observed differences in fecal microbial composition between these populations, suggesting that management-related factors, such as feeding regimes and the degree of human intervention, may be associated with microbiota variation. However, given the observational nature of our data, these relationships should be interpreted with caution, and further studies incorporating controlled dietary and management variables are needed. The observed variation in fecal microbiota among populations suggests several potential considerations for conservation practices. Differences associated with feeding regimes indicate that diet composition and supplementary feeding strategies may influence microbial communities. In addition, geographic variation in microbiota highlights the potential relevance of environmental conditions when planning translocations. Notably, the October samples in this study exhibited characteristics of a transitional state between seasonal groups, suggesting that this period may represent a phase of microbiota restructuring. This observation indicates that the timing of translocation could be considered carefully and that periods of seasonal transition may warrant closer monitoring. Microbiome profiling based on fecal samples may offer a non-invasive approach for characterizing variation in microbial communities following reintroduction. In this study, we observed variation in the relative abundance of certain taxa, including Paeniclostridium, which has been associated with gut microbial imbalance under specific conditions (e.g., host stress, immune suppression, or microbiota imbalance) in previous studies. However, the extent to which such variation can be used to assess population status or health requires further validation.

This study has several limitations. First, fecal microbiota composition is influenced by multiple interacting factors, including climate, diet, water chemistry, soil properties, and management practices, and thus the observed differences cannot be attributed to any single factor. Although paired comparisons were conducted, establishing causal relationships or quantifying the relative contributions of these factors is beyond the scope of this study. Second, while samples were collected across multiple years, limited sample sizes within individual years precluded a formal assessment of interannual variation; therefore, the indication from PCoA that seasonal variation may structure the microbiota should be interpreted with caution. Third, some location-specific background information (e.g., dietary data for the Daqingshan population) was derived from unpublished master’s theses, which, although informative, lack peer review and may reduce the robustness of the supporting evidence. Finally, this study relied exclusively on fecal samples, which provide only an indirect representation of the gastrointestinal microbiota; future opportunistic sampling from deceased individuals may allow for more direct characterization.

## 5. Conclusions

From a microbial ecological perspective, this study provides the first characterization of the fecal microbiota inferred from fecal samples of *E. davidianus* in the Daqingshan National Nature Reserve and highlights its dynamic response to seasonal phenological variation. The observed microbial shifts suggest a degree of ecological flexibility that may facilitate host suitability to the semi-arid grassland environment following reintroduction. Comparative analyses across regions further indicate that fecal microbiota composition may vary under different environmental conditions and management regimes, reflecting context-dependent microbial assembly patterns in reintroduced populations. Our findings provide a scientific basis for formulating conservation and management strategies for *E*. *davidianus* in different geographic regions. However, given the complex and interacting influences of climate, diet, environmental chemistry, and management practices, these interpretations should be treated with caution. The present study does not allow us to disentangle the relative contributions of these factors or establish causal relationships. Future studies integrating dietary analyses, environmental measurements, and controlled designs will be essential to better understand the mechanisms driving fecal microbiota variation in *E. davidianus*.

## Data Availability

The data supporting this study’s findings are available from the corresponding author upon reasonable request.

## Supplementary Materials

The following supporting information can be downloaded at: https://www.mdpi.com/article/10.3390/ani16111698/s1, Figure S1: Diversity of the fecal microbiota in *Elaphurus davidianus* between Inner Mongolia (DQS), Beijing (BJ), and Hubei (SS). (A) Abundance-based coverage estimator. (B) Coverage. (C) Simpson. (B) Principal coordinates analysis (PCoA) plot based on Bray–Curtis distances for three groups; Figure S2: Heatmap of Pathway level 2 of the rainy- and dry-season groups; Table S1: Sample collection information for fecal samples from Père David’s deer in the Daqingshan National Nature Reserve, Inner Mongolia; Table S2: Parasite egg infection status of Père David’s Deer in the Daqingshan National Nature Reserve (2021–2023). Table S3: Summary of sequencing information for fecal samples from Père David’s deer in the Daqingshan National Nature Reserve, Inner Mongolia; Table S4: Alpha diversity indices of the fecal microbiota in Père David’s deer between Inner Mongolia (DQS), Beijing (BJ), and Hubei (SS); Table S5: Alpha diversity indices of the fecal microbiota in Père David’s deer from the Daqingshan National Nature Reserve, Inner Mongolia.
