# Supplementary figures and images for: Phenological Changes in the Fecal Microbiota of Elaphurus davidianus in Inner Mongolia Daqingshan National Nature Reserve
Source: Animals (Basel). 2026 Jun 1;16(11):1698. doi: 10.3390/ani16111698 (PMC13255627; doi:10.3390/ani16111698)

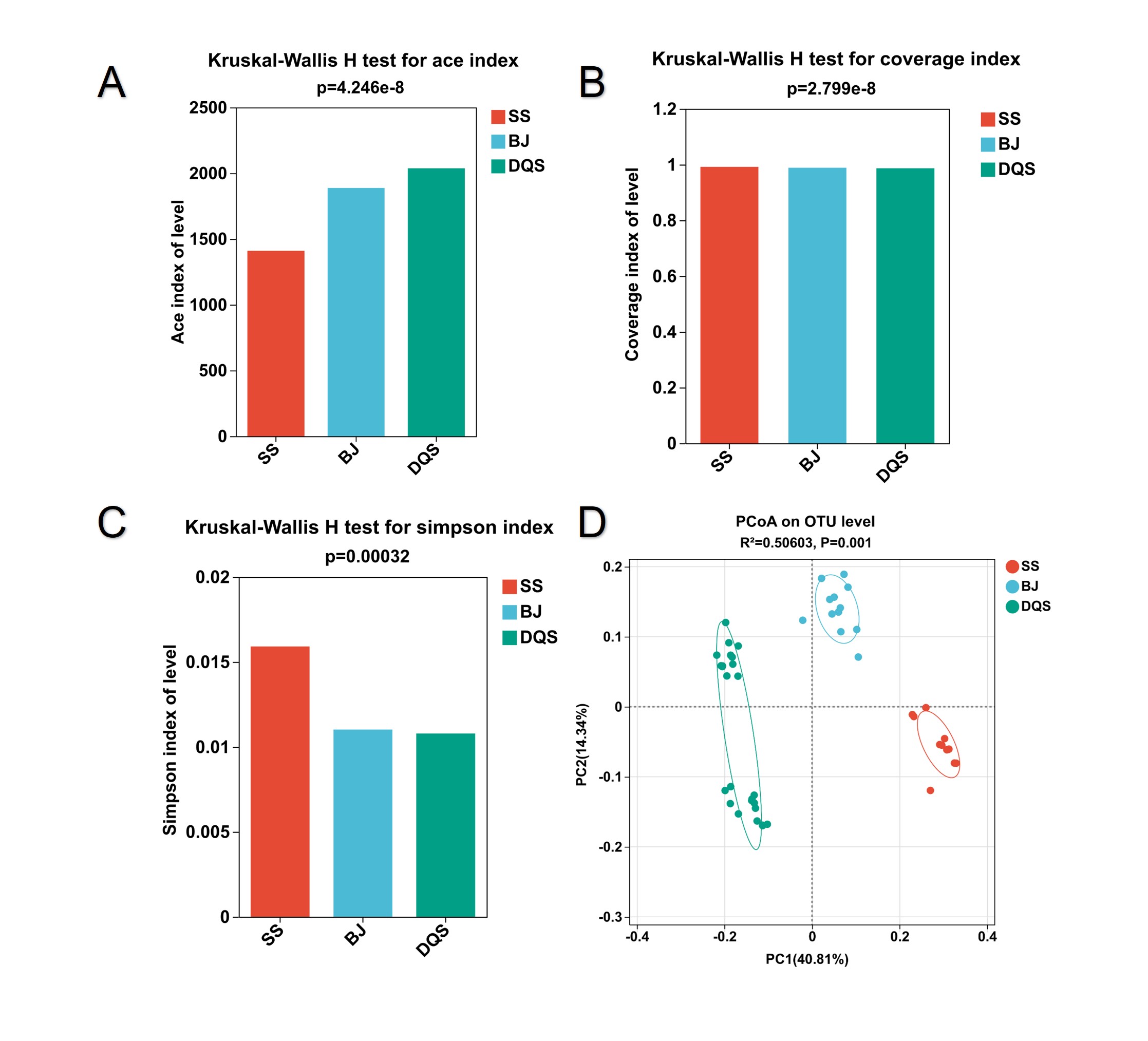

Supplement: Supplementary file 1 [file animals-16-01698-s001.zip › Supplementary_Figure_S1.jpg]
